# Effects of 6-mercaptopurine in pressure overload induced right heart failure

**DOI:** 10.1371/journal.pone.0225122

**Published:** 2019-11-12

**Authors:** Julie Birkmose Axelsen, Stine Andersen, Xiao-Qing Sun, Steffen Ringgaard, Janus Adler Hyldebrandt, Kondababu Kurakula, Marie-José Goumans, Frances S. de Man, Jens Erik Nielsen-Kudsk, Harm-Jan Bogaard, Asger Andersen

**Affiliations:** 1 Department of Cardiology–Research, Aarhus University Hospital, Aarhus, Denmark; 2 Department of Pulmonology, VU University Medical Center, Amsterdam, The Netherlands; 3 MR Centre, Aarhus University Hospital, Aarhus, Denmark; 4 Department of Anesthesiology and Intensive Care, Aarhus University Hospital, Aarhus, Denmark; 5 Department of Cell and Chemical Biology, Leiden University Medical Center, Leiden, The Netherlands; Scuola Superiore Sant'Anna, ITALY

## Abstract

**Background:**

Several antineoplastic drugs have been proposed as new compounds for pulmonary arterial hypertension treatment but many have cardiotoxic side effects. The chemotherapeutic agent 6-mercaptopurine may have an effect in treatment of pulmonary arterial hypertension but at the same time, its effects on the afterload adaption of the right ventricle is unpredictable due to interaction with multiple downstream signalling pathways in the cardiomyocytes. We investigated the direct cardiac effects of 6-mercaptopurine in rats with isolated right heart failure caused by pulmonary trunk banding (PTB).

**Methods:**

Male Wistar rat weanlings (112±2 g) were randomized to sham operation (sham, n = 10) or PTB. The PTB animals were randomized to placebo (PTB-control, n = 10) and 6-mercaptopurine (7.5 mg/kg/day) groups with treatment start before the PTB procedure (PTB-prevention, n = 10) or two weeks after (PTB-reversal, n = 10). Right ventricular effects were evaluated by echocardiography, cardiac MRI, invasive pressure-volume measurements, and histological and molecular analyses.

**Results:**

PTB increased right ventricular afterload and caused right ventricular hypertrophy and failure. 6-mercaptopurine did not improve right ventricular function nor reduce right ventricular remodelling in both prevention and reversal studies compared with placebo-treated rats.

**Conclusion:**

Treatment with 6-mercaptopurine did not have any beneficial or detrimental effects on right ventricular function or remodelling. Our data suggest that treatment of pulmonary arterial hypertension with 6-mercaptopurine is not harmful to the failing right ventricle.

## Introduction

Pulmonary arterial hypertension (PAH) is a rapidly progressive and lethal disease [1] with a prevalence in Europe of approximately 50 patients per million [2, 3]. The disease is characterized by increased resistance of the pulmonary arterioles causing increased right ventricular (RV) afterload. The RV adapts to this increased load via several compensatory mechanisms, but over time these are not sufficient to prevent progression to RV failure, which is the predominant cause of death in PAH patients [4]. While current PAH therapeutics reduce pulmonary vascular resistance, they only partially reverse RV dysfunction. In fact, RV function can further decline even after a reduction in pulmonary vascular resistance [5]. The outcome for PAH patients remains poor with a 3-year survival rate ranging from 55%-74% when treated with current therapeutic regimes [1, 6–8]. Therefore, it is essential to search for new therapeutic agents, which target alternative pathways and which may have direct beneficial effects on RV function.

6-mercaptopurine (6-MP) is a chemotherapeutic agent, which has been used for the treatment of childhood acute lymphoblastic leukaemia and inflammatory bowel diseases for decades [9]. Metabolism and mechanisms of action of 6-MP are still not fully understood [10]. 6-MP exerts its anti-inflammatory effects through inhibition of Rac1 [11]. Other studies have shown that 6-MP increases Nur77 expression and activation in smooth muscle- and endothelial cells, and thereby reduces cell proliferation [12, 13]. 6-MP was therefore considered as a possible new treatment for PAH [14]. The effects of 6-MP on Nur77 expression and activation in cardiomyocytes are, to our knowledge, unknown. Furthermore, the role of Nur77 on the heart is not well understood, and some studies propose a detrimental effect on left ventricular (LV) afterload adaption in cardiac disease [15–18]. One could therefore fear that an increase in Nur77 has an adverse effect on the RV in PAH patients.

A recent study on experimentally induced pulmonary hypertension indicates that 6-MP may have beneficial effects on pulmonary vascular remodelling and the subsequent development of RV failure [14]. Furthermore, azathioprine, a prodrug of 6-MP, has been shown to increase LV ejection fraction (EF) in patients with inflammatory myocarditis and reduce cardiac inflammation, fibrosis, and apoptosis [19–23]. The positive effects seen on the RV could in the first case be secondary effects caused by reduced pulmonary pressures by 6-MP, and in the second case be due to the anti-inflammatory effects of 6-MP. Thus, leaving the effects of 6-MP on pressure overload induced RV failure unknown.

Several antineoplastic drugs have been proposed as new compounds for PAH treatment because of their ability to eliminate excess vascular cells and thereby reduce the causative thickening of the pulmonary vascular wall [24]. Unfortunately, many of these compounds have cardiotoxic side effects [25, 26].

In this study, we therefore aimed to evaluate the direct cardiac effects of 6-MP treatment on RV function and remodelling in rats with pressure overload induced RV failure caused by pulmonary trunk banding (PTB).

## Methods

### Animals

Male Wistar rats (Janiver Labs, Hannover) were given free access to water and standard rat chow (Altromin #1324; Altromin, Lage, Germany). Two animals per cage were housed in a room with a 12-hour light-dark cycle and a temperature of 23°C. The rats were treated according to Danish national guidelines, and all experiments were approved by the Institutional Ethics Review Board, the Danish Animal Experiments Inspectorate, and conducted in accordance with the Danish Law for animal research (authorization number 2016-15-0201-01040, Ministry of Environment and Food of Denmark).

### Study design

**Fig 1:**
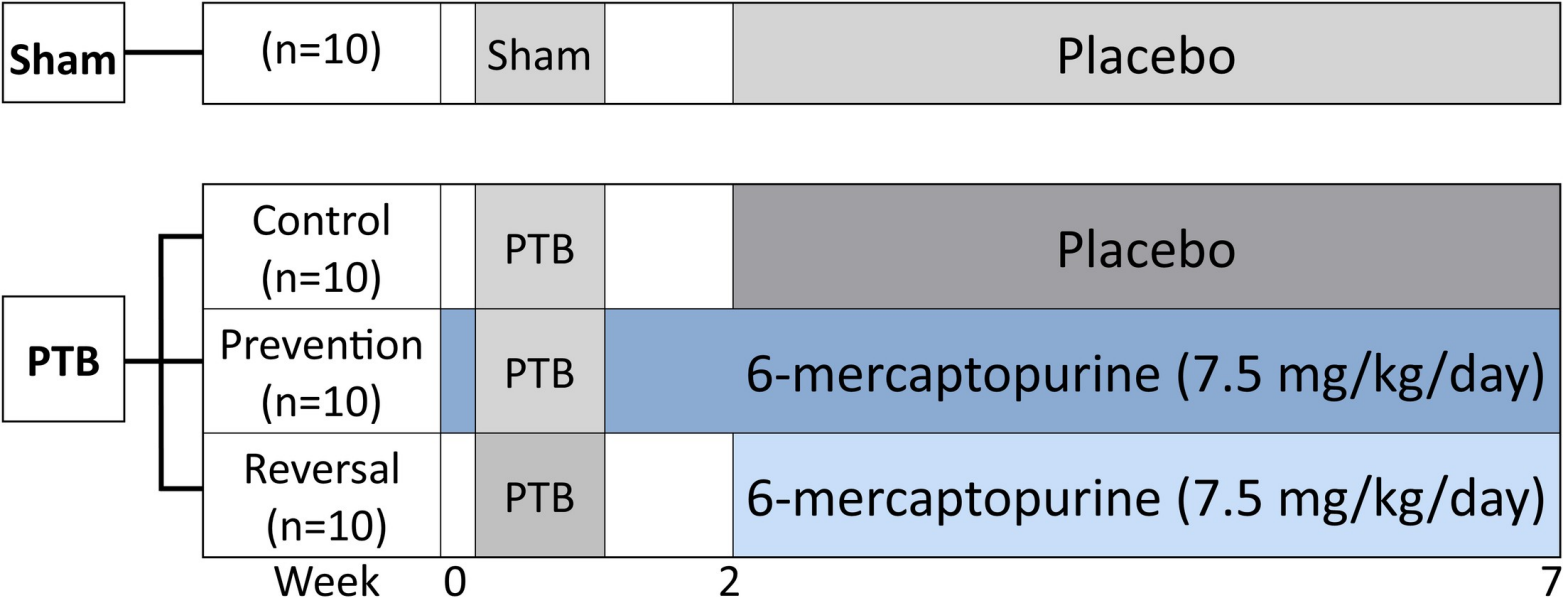
Study design. Male Wistar rats were randomized to sham or pulmonary trunk banding (PTB) operation. The PTB rats were subsequently randomized to either placebo or 6-mercaptopurine treatment. Vehicle or 6-mercaptopurine were given via drinking water from one day before the surgery or two weeks after. Two weeks after the procedure, an echocardiography was performed on all the rats. Seven weeks after the surgery, cardiac function was evaluated by echocardiography, MRI, and invasive pressure-volume measurements and the rats were euthanized.

RV failure was induced by pulmonary trunk banding (PTB). Rats were randomized to one of four groups: a control PTB group (PTB-control, n = 10); a prevention PTB group (PTB-prevention, n = 10); a reversal PTB group (PTB-reversal, n = 10); or sham group (Sham, n = 10). In total, 40 rats were included in this study. The two treatment groups, PTB-prevention and PTB-reversal, received DMSO-dissolved 6-MP (7.5 mg/kg/day) in the drinking water from one day before the surgery or two weeks after the surgery, respectively. In a previous study, the dosage of 7.5 mg/kg/day of 6-MP in the drinking water was proven to be a safe and relevant dosage to use in rats [27]. This dosage is equivalent to the low maintenance dosage of 1.5 mg/kg/day used in patients with chronic bowel disease [28]. The PTB-control and sham groups received placebo (DMSO) treatment from two weeks after the surgery. Two weeks after the surgery, an echocardiography was performed to verify RV dysfunction in the PTB rats. Seven weeks after the surgery, RV function was evaluated by echocardiography, MRI, and invasive pressure-volume measurements. Afterwards, the rats were euthanized, the hearts excised, and histochemical and molecular analyses performed to assess RV remodelling (Fig 1).

### Pulmonary trunk banding

Banding of the pulmonary trunk was performed as described previously [29]. The rats (112 g ± 12 g) were anesthetized with sevoflurane (7% induction, 3.5% maintenance in 2:1 O_2_/air mix), intubated, and ventilated (Abbot Scandinavia, Solona, Sweden; RF 76 min^-1^ and tidal volume 2 mL). The rats were injected with s.c. buprenorphine (0.1 mg/kg, Temgesic, Indivior UK Limited, United Kingdom), shaved on the thorax, and a lateral thoracotomy was performed. The pulmonary trunk was carefully separated from the ascending aorta, and the banding was made with a ligating clip applier modified to compress a titanium clip to a pre-set inner diameter of 0.7 mm. The thorax was closed in three layers, and the rats received s.c. carprofen (5 mg/kg, Norodyl Vet, ScanVet Animal Health, Fredensborg, Denmark) as additional analgesics and 2 mL s.c. isotonic saline solution to compensate for fluid loss. Afterwards the rats were treated with buprenorphine in the drinking water (7.4 ug/mL) for 3 days to relieve postoperative pain. Sham operated animals underwent the same procedure except for the application of the clip around the pulmonary trunk.

### Evaluation of hemodynamic and anatomic measures

RV dimensions and function were assessed by echocardiography, MRI, and invasive pressure-volume measurements (S1 Appendix). The heart was quickly excised and the RV separated from the LV + septum and weighed. RV/tibia length (TL) was used as a measure of RV hypertrophy. RV tissue was snap frozen for molecular analyses and immersion fixated in formalin 4% for histology. For estimation of ascites and pleural fluid a gaze swap was weighed before and after swiping the thoracic and abdominal cavities and a surplus of >1g was used as a cut off. Remaining organs were weighed, and the liver examined for dark discoloration (nutmeg liver) as a sign of backward failure. A small amount of blood was used for quantification of white blood cell count, haematocrit, and red blood cell count using a hematology analyser (Sysmex KX-21N). Methods for histology, quantitative real-time polymerase chain reaction (PCR), western blotting, nuclear and cytoplasmic fractioning of tissue lysates, and immunofluorescence are described in S1 Appendix. Except for histology, we chose only to analyse sham, PTB-control, and PTB-reversal group, as the PTB-reversal group is clinically more relevant than the PTB-prevention group.

### Statistics

Statistical analyses were performed using GraphPad Prism 7.04 for Windows (GraphPad Software, La Jolla California, USA, www.graphpad.com). All data were tested for normal distribution by QQ-plots and Brown-Forsythe test and non-parametric tests were used if data was not normally distributed. Normally distributed quantitative data are expressed as mean ± standard error of mean (SEM). Non-normally distributed data was transformed and is presented by box plots. Analyses were performed using one-way ANOVA with Bonferroni post-hoc comparison or a non-parametric Kruskal-Wallis test of selected groups to evaluate the PTB model (PTB-control vs sham), the preventive effects of 6-MP (PTB-control vs PTB-prevention), and the reversal effects of 6-MP (PTB-control vs PTB-reversal). Dichotomous outcomes were compared between groups by Fisher’s exact test. P<0.05 were considered significant.

## Results

### Effects of pulmonary trunk banding (PTB)

**Fig 2:**
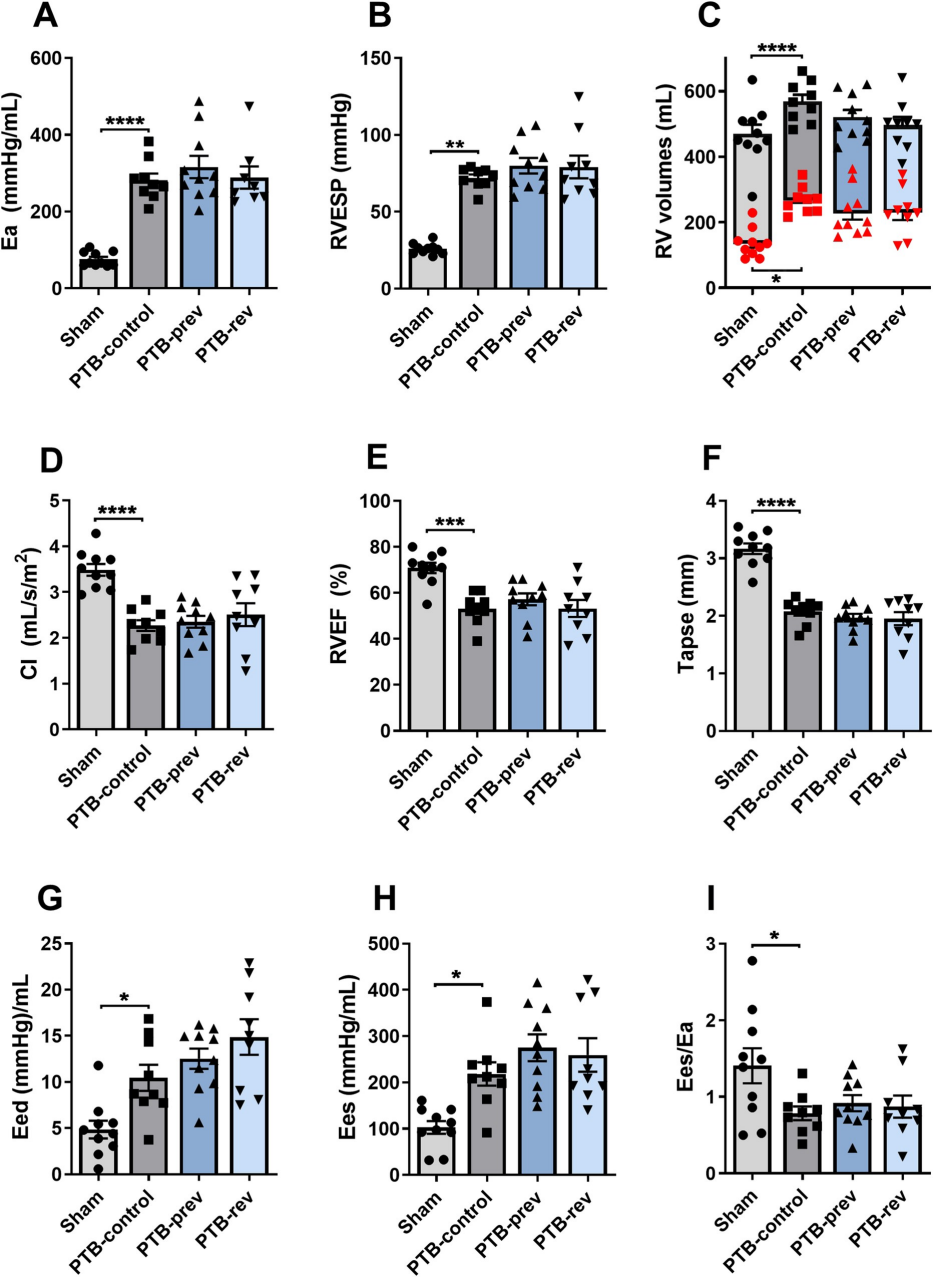
Effects of pulmonary trunk banding and 6-mercaptopurine treatment on right ventricular function at the end of the study. PTB: Pulmonary trunk banding; prev: Prevention group receiving 6-mercaptopurine treatment from one day before surgery; rev: Reversal group receiving 6-mercaptopurine treatment from two weeks after surgery. (A) Arterial elastance. (B) Right ventricular (RV) systolic pressure. (C) RV end-systolic (red dots) and end-diastolic volumes (black dots). (D) Cardiac index. (E) RV ejection fraction. (F) Tricuspid annular plane systolic excursion. (G) End-diastolic elastance. (H) End-systolic elastance. (I) Ventriculo-arterial coupling. Results are expressed as scatterplots with mean ± SEM. *P<0.05; ***P<0.001; ****P<0.0001.

PTB-control rats were compared with sham-operated rats to assess the effects of the PTB procedure (Table 1, Fig 2). PTB increased RV afterload (arterial elastance (Ea)) and RV end-systolic pressures (RVESP) in PTB-control compared with sham rats. Further, we observed increased RV volumes indicating dilatation of the RV in the PTB rats. The PTB-control rats had signs of RV dysfunction evident by decreased cardiac index (CI), RV ejection fraction (EF), and tricuspid annular plane systolic excursion (TAPSE). Invasive pressure-volume measurements showed increased RV end-diastolic elastance (Eed) in PTB-controls compared with sham indicating diastolic dysfunction. RV end-systolic elastance (Ees), a measure of RV contractility, was increased in the PTB rats but not sufficient to maintain the ventriculo-arterial coupling measured by Ees/Ea. Two animals died prematurely during the study with signs of right heart failure, one from the PTB-control group and one from the PTB-reversal group, without any significant differences in mortality between the groups. The analyses of the different groups, therefore, consisted of 10 sham, 9 PTB-control, 10 PTB-prevention, and 9 PTB-reversal animals.

**Table 1:** Anatomic and hemodynamic data at the end of the study.

	Control	PTB		
	Sham n = 10	PTB-control n = 9	PTB-prevention n = 10	PTB-reversal n = 9
**Anatomical data**				
Body weight (g)	390±6	394±13	381±7	354±13^
RV (g)	0.20±0.01	0.49±0.01****	0.47±0.01	0.44±0.03
LV+S (g)	0.78±0.01	0.86±0.03	0.92±0.03	0.86±0.04
LV CSA μm^2^	630±20	664±22	656±16	657±19
RV/(LV+S)	0.25±0.004	0.57±0.02****	0.51±0.01	0.52±0.02
TL (mm)	41.0±0.21	40.7±0.41	40.0±0.11	39.8±0.36
Lungs (g)	1.36±0.03	1.46±0.05	1.45±0.05	1.38±0.04
Liver (g)	14.3±0.54	13.4±0.62	14.1±0.46	12.7±0.09
Kidneys (g)	2.35±0.03	2.33±0.10	2.16±0.05	2.10±0.09
Spleen (g)	1.01±0.04	0.97±0.05	0.84±0.04	0.85±0.04
Extracardiac manifestations	0%	0%	20%	10%
**Hematology**				
WBC (∙10^9^/L)	9.48±0.76	7.58±0.81	6.65±0.58	4.09±0.76^^
Hematokrit (L/L)	0.44±0.01	0.48±0.01	0.44±0.01	0.42±0.02
RBC (∙10^9^/L)	7.95±0.18	8.50±0.21	8.06±0.22	7.60±0.41
**Hemodynamic measures**				
HR (bpm)	327±12	289±6	303±9	298±13
RV SV (μL)	354±15	264±14***	261±9	245±15
RV diastolic pressure (mmHg)	7.38±1.04	6.57±0.65	5.19±0.46	5.58±0.90
RV filling pressure	2.23±0.16	3.74±0.44*	4.56±0.52	4.40±0.48
MAP (mmHg)	112±5	114±4	119±3	114±5
RV dP/dt max (mmHg/s)	1248±90	2546±121****	2822±181	2798±266
RV dP/dt min (mmHg/s)	-1090±83	-2573±156****	-2794±135	-2721±202

RV: Right ventricle; LV+S: Left ventricle + septum; LV CSA: Left ventricle cross sectional area; TL: Tibia length; Extracardiac manifestations: nutmeg liver, ascites and/or hydrothorax; WBC: White blood cell count; RBC: Red blood cell count; HR: Heart rate; SV: Stroke volume; MAP: Mean arterial pressure; dP/dt max: First derivative (maximal) of right ventricular systolic pressure; dP/dt min: First derivative (minimal) of right ventricular systolic pressure.

Data are presented as mean ± SEM.

*P<0.05

***P<0.001

****P<0.0001; PTB-control vs. sham.

^^P<0.01; 6-MP treatment vs. PTB-control.

**Fig 3:**
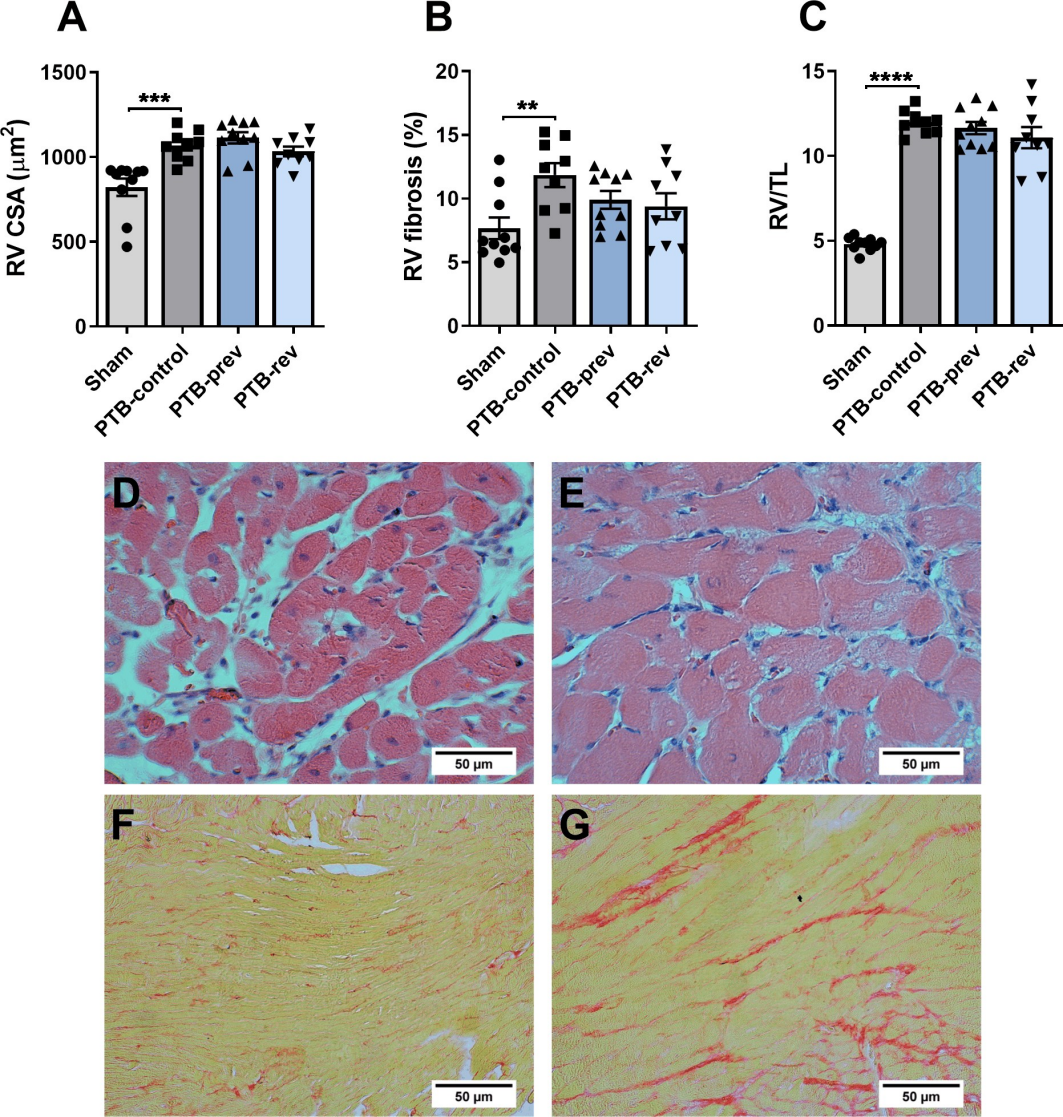
Effects of pulmonary trunk banding and 6-mercaptopurine treatment on anatomy and histology. PTB: Pulmonary trunk banding; prev: Prevention group receiving 6-mercaptopurine treatment from one day before surgery; rev: Reversal group receiving 6-mercaptopurine treatment from two weeks after surgery. (A) Right ventricular (RV) cardiomyocyte cross sectional area. (B) RV fibrosis. (C) RV weight/tibia length. Histological sections stained with hematoxylin and eosine from (D) Sham and (E) Pulmonary trunk banding (PTB) control group and sections stained with picrosirius red from (F) Sham and (G) PTB-control. Results are expressed as scatterplots with mean ± SEM and are analysed by comparing PTB-control vs sham, PTB-control vs PTB-prevention, and PTB-control vs PTB-reversal.**P<0.01; ***P<0.001; ****P<0.0001.

**Fig 4:**
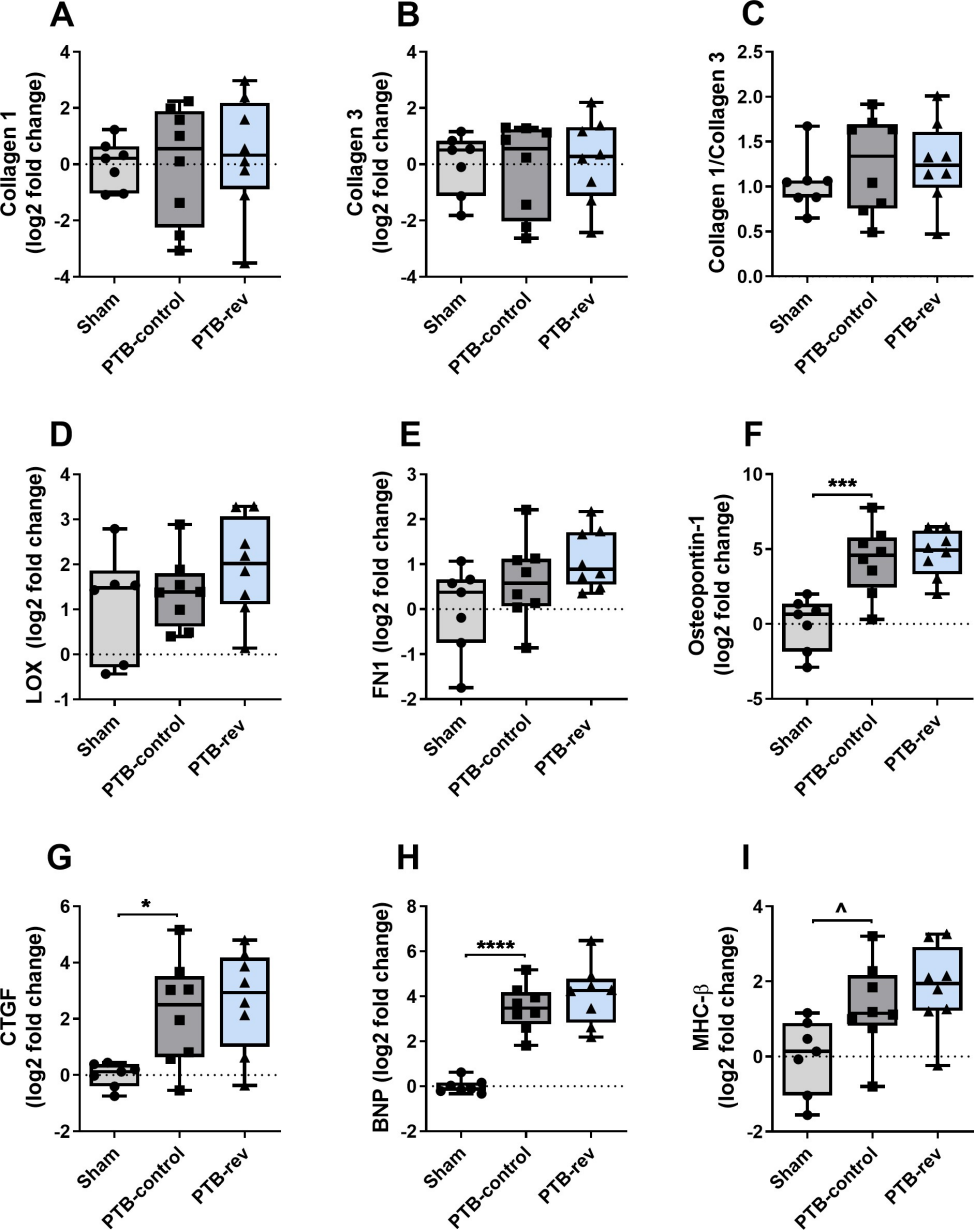
Effects of pulmonary trunk banding and 6-mercaptopurine treatment on fibrosis, hypertrophy, and heart failure markers. Gene expression levels quantified by real-time polymerase chain reaction (real-time PCR) and normalized to glyceraldehyde 3-phosphate dehydrogenase (GAPDH). Results presented by box plots. PTB: Pulmonary trunk banding; rev: Reversal group receiving 6-mercaptopurine treatment from two weeks after surgery. (A) mRNA expression of collagen 1. (B) mRNA expression of collagen 3a. (C) Collagen 1/collagen 3a ratio. (D) mRNA expression of osteopontin-1. (E) mRNA expression of connective tissue growth factor (CTGF). (F) mRNA expression of lysyl oxidase (LOX). (G) mRNA expression of fibronectin 1 (FN1). (H) mRNA expression of brain natriuretic peptide (BNP). (I) mRNA expression of myosin heavy chain-β (MHC-β).^P = 0.058; *P<0.05; ***P<0.001; ****P<0.0001.

**Fig 5:**
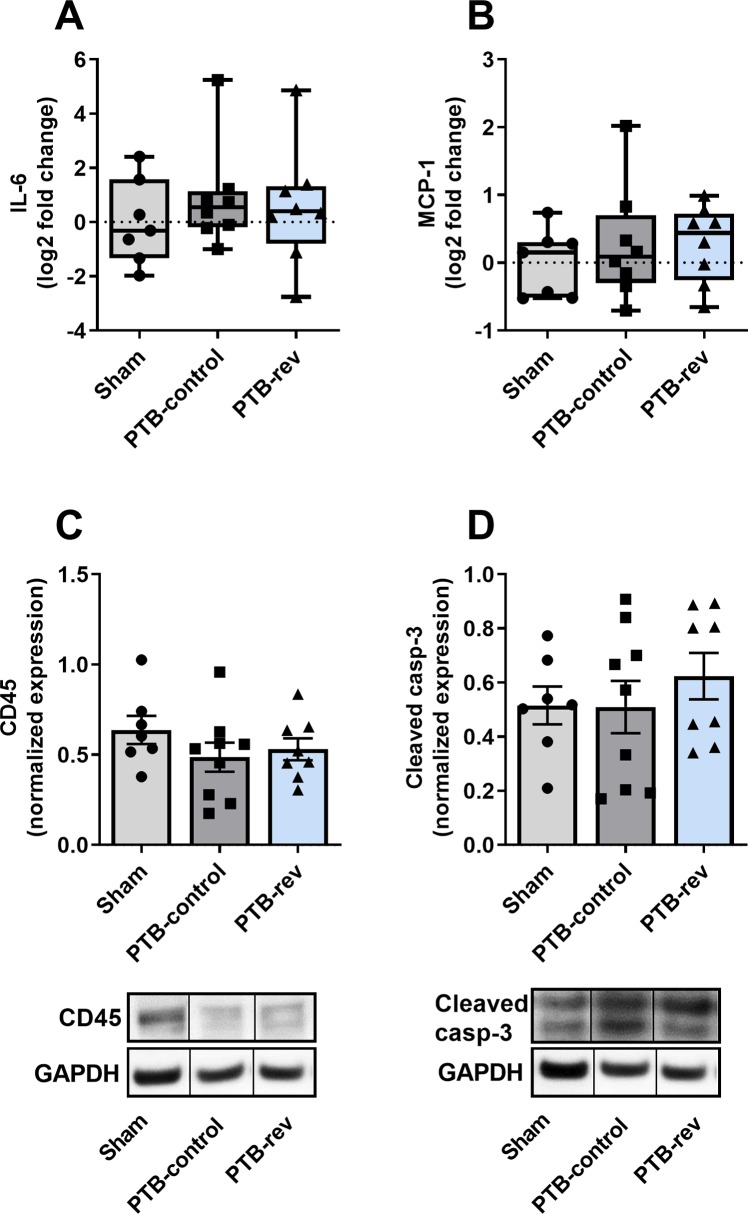
Effects of pulmonary trunk banding and 6-mercaptopurine treatment on inflammation and apoptosis in the right ventricle. Gene expression levels of interleukin 6 (IL-6) and monocyte chemotactic protein 1 (MCP-1) quantified by real-time polymerase chain reaction (real-time PCR) and normalized to glyceraldehyde 3-phosphate dehydrogenase (GAPDH). Results presented by box plots. Western blot analyses of CD45 and cleaved caspase-3 normalized to GAPDH. Results expressed as scatterplots with mean ± SEM. PTB: Pulmonary trunk banding; rev: Reversal group receiving 6-mercaptopurine treatment from two weeks after surgery.(A) mRNA expression of IL-6. (B) mRNA expression of MCP-1. (C) Protein expression of CD45 with representative lanes from a single blot. (D) Protein expression of cleaved caspase-3 with representative lanes from a single blot.

**Fig 6:**
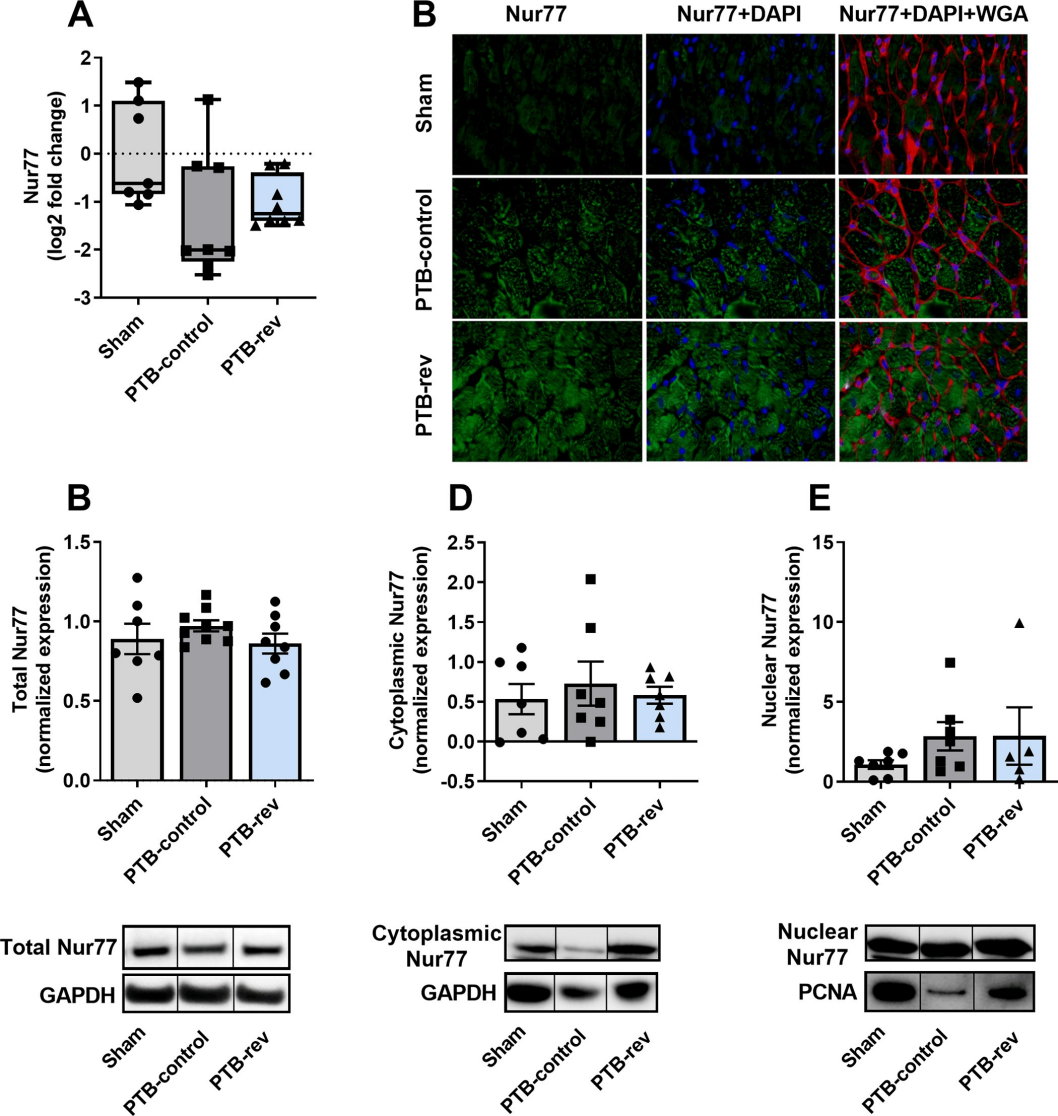
Effects of pulmonary trunk banding and 6-mercaptopurine treatment on Nur77 expression in the right ventricle. Gene expression levels of Nur77 quantified by real-time polymerase chain reaction (real-time PCR) and normalized to glyceraldehyde 3-phosphate dehydrogenase (GAPDH) presented by box plots. Western blot analyses of total Nur77 normalized to GAPDH and Nur77 expression in isolated cytoplasmic and nuclear fraction normalized to GAPDH and proliferating cell nuclear antigen (PCNA), respectively. Results are expressed as scatterplots with mean ± SEM and with representative lanes from a single blot. PTB: Pulmonary trunk banding; rev: Reversal group receiving 6-mercaptopurine treatment from two weeks after surgery.(A) mRNA expression of Nur77. (B) Protein expression of total Nur77. (C) Representative images of immunofluorescence staining for Nur77 (green), DAPI (blue), and Wheat Germ Agglutinin (WGA; red). (D) Protein expression of Nur77 from cytoplasmic fraction. (E) Protein expression of Nur77 from nuclear fraction.

The increased RV pressures caused RV hypertrophy as seen by increased RV weight normalized to tibia length (RV/TL) in the PTB-control group compared with sham rats. Histology revealed an increase in RV cardiomyocyte size and fibrosis in the model (Fig 3). To explore the potential mechanisms for the increased fibrosis, we analyzed genes related to collagen production. The analyses showed no changes in myocardial mRNA expression levels of collagen 1, collagen 3a, lysyl oxidase (LOX), or fibronectin-1 (FN1). However, gene expression levels of the two profibrotic proteins, osteopontin-1 and connecting tissue growth factor (CTGF), were increased in the PTB rats. An increase in gene expression of the heart failure marker, brain natriuretic peptide (BNP), and a trend towards increased myosin heavy chain-β (MHC-β), a hypertrophy marker, were found in the PTB-control group compared with sham rats (Fig 4). There were no signs of inflammation of the RV in the PTB-control group compared with sham rats assessed by gene expression levels of interleukin 6 (IL-6) and monocyte chemotactic protein 1 (MCP-1) or by protein expression of CD45. Protein expression levels of cleaved caspase-3 showed no sign of increased apoptosis in the model (Fig 5). When looking at the mechanisms of Nur77, there were no difference in either gene or protein expression levels of total Nur77 nor in protein expression of Nur77 in isolated fractions from cytoplasma and nucleus. However, on the immunofluorescence stainings, the Nur77 protein expression seemed increased in the PTB-control group compared with the sham group (Fig 6).

### Effects of 6-MP treatment on RV function

To evaluate the effects of treatment with 6-MP the two treatment groups, PTB-prevention and PTB-reversal, were compared with the PTB-control group (Table 1, Fig 2). At baseline, 6-MP treated rats showed no differences in either weight or echocardiographic parameters compared with PTB-control (Table B in S1 Appendix). At evaluation, no differences in RV function were found between the PTB-control group and the two 6-MP treated groups assessed by CI, EF, and TAPSE. Neither did RV volumes differ between the groups. 6-MP-treatment did not show any effects on either RVESP, RV stiffness (Eed), RV contractility (Ees), or ventriculo-arterial coupling (Ees/Ea). There were no differences in systemic mean blood pressure (MAP) when comparing the 6-MP groups with PTB-control.

Looking at RV hypertrophy, treatment with 6-MP did not reduce the RV/TL ratio or the RV/(LV + septum) weight ratio and 6-MP did not have any effects on cardiomyocyte cross sectional area (CSA) or fibrosis when compared with PTB-control (Table 1, Fig 3). Further molecular analyses of the effects of 6-MP on fibrosis and collagen production in the PTB-reversal group did not reveal any changes in mRNA levels of collagen 1, collagen 3a, CTGF, osteopontin-1, LOX, or FN1. Analyses of genes related to RV hypertrophy and failure showed no changes in levels of MHC-β or BNP mRNA expression with 6-MP treatment compared with PTB-control (Fig 4). The PTB reversal group did not differ from the PTB-control group regarding gene or protein expression levels related to inflammation or apoptosis (Fig 5). There were no differences in expression levels of cellular Nur77. Immunofluorescence images showed increased Nur77 in the nucleus of cardiac cells of PTB-reversal rats compared with PTB-control, but western blot analysis of the protein expression levels of Nur77 in cytoplasmic and nuclear fractions did not confirm this finding (Fig 6). The PTB-reversal group showed reduced end-body weight and white blood cell count compared with the PTB-control group (Table 1).

## Discussion

This study showed that: 1) PTB caused RV failure evident by decreased RV function and adverse remodeling and 2) Treatment with 6-MP did not influence RV function or remodeling compared with placebo.

### Effects of PTB

#### RV failure in PTB

The PTB procedure caused increased RV afterload and RVESP compared with sham operation. Hemodynamic measures of the PTB-rats showed signs of RV dysfunction with decreased cardiac index and reduced RV EF caused by a reduced longitudinal shortening of the ventricle (TAPSE). This correlates with RV failure development in PAH patients [5, 30, 31]. The PTB-rats developed compensated RV failure as only few of the animals showed extra-cardiac manifestations including nut-meg liver, ascites, or pleural fluid. In PAH patients, fluid retention is one of the cardinal symptoms of RV failure [32]. As an expected result of the chronic increase in afterload, the RV contractility (Ees) was increased in PTB-control rats compared with sham. However, the increase in contractility was not sufficient to maintain the ventriculo-arterial coupling, confirming that the RV of the PTB rats failed to compensate for the increase in afterload, which is consistent with data from RV failure patients [30].

#### RV remodelling in PTB

In PTB-rats, we saw RV hypertrophy and increased fibrosis, which have also been demonstrated in RV biopsies from patients with PAH [33, 34] and in models of experimentally induced pulmonary hypertension including the Sugen-Hypoxia and the monocrotaline model [35, 36]. Regarding RV hypertrophy, we saw a clear increase in cardiomyocyte size in PTB-control compared with sham. Increased gene expressions of MHC-β and BNP confirmed the presence of RV hypertrophy and failure on molecular levels in the PTB-control group. In PAH patients, a shift from MHC-α to β is associated with reduced RV contractile function [33], while increased plasma BNP levels are associated with the degree of RV dysfunction and mortality [37].

We found increased fibrosis in the PTB-control group compared with sham. An increase in fibrosis may be due to increased synthesis, enhanced cross-linking, or decreased degradation of the collagen fibers. Inconsistent with previous PTB studies [38], mRNA expression levels of collagen 1 or 3a were not increased in this study. These results, however, could have been influenced by the dispersion of the expression levels as seen by the width of bars in Fig 4A–4C. LOX contributes to cross binding of the collagen filaments increasing the stiffness, while FN1 is a facilitator of LOX [39, 40]. Osteopontin-1 and CTGF are known profibrotic mediators, additionally, osteopontin-1 reduces breakdown of collagen [41, 42]. We did not see any changes in LOX or FN1 gene levels but osteopontin-1 and CTGF mRNA expression levels were increased in the PTB-control group compared with sham. Therefore, the increased fibrosis development in this study is supposedly caused by reduced collagen turnover evident by increased osteopontin-1 and CTGF gene levels. A certain level of fibrosis might be beneficial in response to pressure overload as it provides mechanical support to cardiomyocytes, and as long as fibrosis is mild will not lead to excessive RV myocardial stiffness [43–47]. Another function of the fibroblasts is to contribute to myocardial function by releasing paracrine factors contributing to hypertrophy [48, 49]. However, it has not been possible to determine whether a ‘threshold’ level of RV fibrosis exists, above which fibrosis becomes detrimental and drives RV failure [45, 46]. In this study, the increased RV fibrosis may contribute to increased mechanical support and hypertrophy improving contractility but on the other hand contribute to diastolic dysfunction by increasing RV stiffness evident by increased Eed. We did not see any changes in gene or protein expression levels of Nur77 in the PTB-control group compared with sham. This is inconsistent with other studies, where the expression of Nur77 in the LV increases in response to different cardiac stressors and in some cases has a detrimental effect on the LV [15–18, 50–52]. Nur77 may not be affected in this model of pressure overload induced RV failure but its response and effect in decompensated rats or other types of RV failure are still unknown.

#### Inflammation and apoptosis in PTB

The role of inflammation and apoptosis on development of RV failure in PAH patients are not well understood [53, 54]. Clinical studies with PAH patients have shown that increased levels of IL-6 are related to worse NYHA functional class [55]. In this model, we did not see an increase in inflammation measured by expression levels of IL-6, MCP-1, and CD45. To our knowledge, this is the first study to investigate inflammation in compensated RV failure in the PTB model. Recently, a study showed increased inflammation in decompensated RV failure in PTB animals [56]. In the monocrotaline rat model, inflammation is only present in the progressive but not in the stable pulmonary hypertension [57]. Together with our results, this insinuates that inflammation is present only in decompensated and not compensated RV failure. Increased apoptosis in PTB-rats have previously been demonstrated in compensated RV failure [54], this is inconsistent with our results as we did not see any changes in cleaved caspase-3 protein levels.

The PTB model caused RV hypertrophy and failure similar to the failing RV in patients with PAH. With this model, it is possible to study the molecular events underlying RV remodeling, which is highly beneficial when searching for new PAH treatments with cardioprotective effects [58].

### Effects of 6-MP treatment

At end-experiment, most measured parameters were consistent between the two treatment groups, PTB-prevention and PTB-reversal, when compared with PTB-control. A reduced white blood cell (WBC) count was found in the PTB-reversal group compared with PTB-control indicating a side effect from 6-MP treatment. Treatment with 6-MP did not alter the hemodynamics of the RV in the rats.

#### The effects of 6-MP on the RV

A recent study on rats with Sugen-hypoxia induced pulmonary hypertension and RV failure showed that 6-MP treatment reduced pulmonary vascular resistance. Furthermore, it improved cardiac function evident by a decrease in RV remodelling and an increase in stroke volume [14]. The animal model used was a non-fixed afterload model making it difficult to separate direct cardiac effects from secondary effects caused by reduced pulmonary pressures. PTB, on the other hand, is a fixed-afterload model of RV failure, which allows for separate evaluation of the direct cardiac effects of 6-MP. With PTB, we saw no beneficial or adverse cardiac effects with 6-MP treatment on isolated RV failure.

#### The mechanisms of 6-MP in RV remodelling

Treatment with 6-MP did not reduce RV remodeling examined by hypertrophy and fibrosis development. In our study, the PTB-rats did not show signs of inflammation, which prevented investigation of the anti-inflammatory effects of 6-MP on the RV. Treatment with 6-MP did not increase apoptosis measured by cleaved caspase-3 supporting that 6-MP does not have cardiotoxic side effects. As there were no changes in gene or protein expression levels of Nur77 in the PTB-reversal group compared with PTB-control, our study suggests that 6-MP does not alter Nur77 expression or activation in RV cardiomyocytes from rats with RV failure. This is consistent with Pires et al [13], who propose that 6-MP may increase Nur77 activity in some but not all cell types [13, 59–61]. From this study, the potentially beneficial but also adverse effects of Nur77 on the RV remain elusive.

The anti-inflammatory effect of 6-MP in high-dosages mainly consist of incorporating purine antagonists in immune cells and in low-dosages inhibiting Rac1 activity in endothelial cells [9, 11, 59, 62]. Since leukopenia and bone marrow suppression are common side effects of treatment with 6-MP we measured WBC and red blood cell count [62, 63]. A recent study found no evidence of 7.5 mg/kg 6-MP affecting the composition of blood cells in rats [14]. In our study, we saw a reduction in WBC count in the PTB-reversal group compared with PTB-control. As we did not see the same pattern in the PTB-prevention group, we primarily see this as a different reaction to the PTB operation causing a weight difference between the two animals in a cage in the PTB-reversal group leading to a slight overdose of the smallest rat. We still consider a dosage of 7.5 mg/kg 6-MP to be safe in rats.

In summary, even with current treatment options PAH remains a progressive and lethal disease [64]. Therefore, it is still important to look for new treatment options for these patients. Several other chemotherapeutic drugs tested in PAH later showed cardiotoxic side effects warranting the need to investigate for cardiotoxic side effects in new drugs [25, 26]. It is therefore worth noticing that we did not see any adverse or toxic cardiac effects with 6-MP treatment with a dosage of 7.5 mg/kg/day. The only potential side effect we saw with 6-MP treatment was a reduction in WBC count in the PTB-reversal group compared with PTB-control. Inconsistent with the azathioprine studies and the recent study on experimentally induced PAH, we did not see any beneficial cardiac effects with 6-MP treatment [14, 19–23]. This discordance might be explained by the importance of the anti-inflammatory effects by 6-MP and the improvement of RV afterload by reducing smooth muscle cell proliferation in the lung vasculature in the PAH model.

### Strengths and limitations

Outbred male Wistar rats were used in this study and the difference between rats and humans should be taken into consideration before clinical translation. All hemodynamic measures were performed in anesthetized animals, which could blunt the difference between the groups. To minimize this problem we carefully followed a well-tested protocol of anesthesia according to previous recommendations [65]. The PTB model caused RV failure similar to the failing RV in patients with PAH making it possible to study the molecular events underlying RV remodeling. In the PTB-reversal group, we observed a weight difference between the two co-caging animals, potentially causing a slight overdose of the smallest rat as the 6-MP concentration in their shared drinking water was adjusted to the weight of the heaviest rat.

## Conclusion

Treatment with 6-MP did not influence RV function nor reduce RV remodeling in rats with pressure overload RV failure induced by PTB. Our study supports that treatment with 6-MP has no toxic or adverse effects on the failing RV. However, we did see a reduction in WBC count, which could be a side effect from 6-MP treatment, and caution is required despite unchanged RV function.

## Supporting information

S1 Appendix Supplementary methods and results. (DOCX)

S1 Dataset Data and image examples. (XLSX)

## References

[1] Humbert M, Sitbon O, Chaouat A, Bertocchi M, Habib G, Gressin V, et al Survival in Patients With Idiopathic, Familial, and Anorexigen-Associated Pulmonary Arterial Hypertension in the Modern Management Era. Circulation. 2010;122(2):156–63. 10.1161/CIRCULATIONAHA.109.911818 20585011

[2] Peacock AJ, Murphy NF, McMurray JJ, Caballero L, Stewart S. An epidemiological study of pulmonary arterial hypertension. The European respiratory journal. 2007;30(1):104–9. 10.1183/09031936.00092306 17360728

[3] Humbert M, Sitbon O, Chaouat A, Bertocchi M, Habib G, Gressin V, et al Pulmonary arterial hypertension in France: results from a national registry. Am J Respir Crit Care Med. 2006;173(9):1023–30. 10.1164/rccm.200510-1668OC 16456139

[4] van de Veerdonk MC, Marcus JT, Westerhof N, de Man FS, Boonstra A, Heymans MW, et al Signs of Right Ventricular Deterioration in Clinically Stable Patients With Pulmonary Arterial Hypertension. Chest. 2015;147(4):1063–71. 10.1378/chest.14-0701 25376008

[5] van de Veerdonk MC, Kind T, Marcus JT, Mauritz G-J, Heymans MW, Bogaard H-J, et al Progressive Right Ventricular Dysfunction in Patients With Pulmonary Arterial Hypertension Responding to Therapy. Journal of the American College of Cardiology. 2011;58(24):2511–9. 10.1016/j.jacc.2011.06.068 22133851

[6] Kane GC, Maradit-Kremers H, Slusser JP, Scott CG, Frantz RP, McGoon MD. Integration of clinical and hemodynamic parameters in the prediction of long-term survival in patients with pulmonary arterial hypertension. Chest. 2011;139(6):1285–93. 10.1378/chest.10-1293 21071530

[7] Thenappan T, Shah SJ, Rich S, Tian L, Archer SL, Gomberg-Maitland M. Survival in pulmonary arterial hypertension: a reappraisal of the NIH risk stratification equation. The European respiratory journal. 2010;35(5):1079–87. 10.1183/09031936.00072709 20032020 PMC8782564

[8] Benza RL, Miller DP, Barst RJ, Badesch DB, Frost AE, McGoon MD. An Evaluation of Long-term Survival From Time of Diagnosis in Pulmonary Arterial Hypertension From the REVEAL Registry. Chest. 2012;142(2):448–56. 10.1378/chest.11-1460 22281797

[9] Aarbakke J, Janka-Schaub G, Elion GB. Thiopurine biology and pharmacology. Trends in Pharmacological Sciences. 1997;18(1):3–7. 10.1016/s0165-6147(96)01007-3 9114722

[10] Innocenti F, Danesi R, Bocci G, Fogli S, Di Paolo A, Del Tacca M. Metabolism of 6-mercaptopurine in the erythrocytes, liver, and kidney of rats during multiple-dose regimens. Cancer chemotherapy and pharmacology. 1999;43(2):133–40. 10.1007/s002800050873 9923818

[11] Marinkovic G, Kroon J, Hoogenboezem M, Hoeben KA, Ruiter MS, Kurakula K, et al Inhibition of GTPase Rac1 in endothelium by 6-mercaptopurine results in immunosuppression in nonimmune cells: new target for an old drug. Journal of immunology (Baltimore, Md: 1950). 2014;192(9):4370–8. 10.4049/jimmunol.1302527 24670805

[12] Wansa KD, Harris JM, Yan G, Ordentlich P, Muscat GE. The AF-1 domain of the orphan nuclear receptor NOR-1 mediates trans-activation, coactivator recruitment, and activation by the purine anti-metabolite 6-mercaptopurine. The Journal of biological chemistry. 2003;278(27):24776–90. 10.1074/jbc.M300088200 12709428

[13] Pires NM, Pols TW, de Vries MR, van Tiel CM, Bonta PI, Vos M, et al Activation of nuclear receptor Nur77 by 6-mercaptopurine protects against neointima formation. Circulation. 2007;115(4):493–500. 10.1161/CIRCULATIONAHA.106.626838 17242285

[14] Kurakula K, Sun X-Q, Happé C, da Silva Goncalves Bos D, Szulcek R, Schalij I, et al 6-mercaptopurine, an agonist of Nur77, reduces progression of pulmonary hypertension by enhancing BMP signalling. European Respiratory Journal. 2019:1802400 10.1183/13993003.02400-2018 31273046

[15] Medzikovic L, Schumacher CA, Verkerk AO, van Deel ED, Wolswinkel R, van der Made I, et al Orphan nuclear receptor Nur77 affects cardiomyocyte calcium homeostasis and adverse cardiac remodelling. Scientific reports. 2015;5:15404 10.1038/srep15404 26486271 PMC4613907

[16] Yan G, Zhu N, Huang S, Yi B, Shang X, Chen M, et al Orphan Nuclear Receptor Nur77 Inhibits Cardiac Hypertrophic Response to Beta-Adrenergic Stimulation. Mol Cell Biol. 2015;35(19):3312–23. 10.1128/MCB.00229-15 26195821 PMC4561731

[17] Wang RH, He JP, Su ML, Luo J, Xu M, Du XD, et al The orphan receptor TR3 participates in angiotensin II-induced cardiac hypertrophy by controlling mTOR signalling. EMBO molecular medicine. 2013;5(1):137–48. 10.1002/emmm.201201369 23197407 PMC3569659

[18] You X, Guo ZF, Cheng F, Yi B, Yang F, Liu X, et al Transcriptional up-regulation of relaxin-3 by Nur77 attenuates beta-adrenergic agonist-induced apoptosis in cardiomyocytes. The Journal of biological chemistry. 2018;293(36):14001–11. 10.1074/jbc.RA118.003099 30006349 PMC6130952

[19] Lu C, Qin F, Yan Y, Liu T, Li J, Chen H. Immunosuppressive treatment for myocarditis: a meta-analysis of randomized controlled trials. Journal of Cardiovascular Medicine. 2016;17(8):631–7. 10.2459/JCM.0000000000000134 25003999

[20] Wojnicz R, Nowalany-Kozielska E, Wojciechowska C, Glanowska G, Wilczewski P, Niklewski T, et al Randomized, placebo-controlled study for immunosuppressive treatment of inflammatory dilated cardiomyopathy: two-year follow-up results. Circulation. 2001;104(1):39–45. 10.1161/01.cir.104.1.39 11435335

[21] Frustaci A, Chimenti C, Russo MA. Randomized study on the efficacy of immunosuppressive therapy in patients with virus-negative inflammatory cardiomyopathy: the TIMIC study. European heart journal. 2009;30(16):1995–2002. 10.1093/eurheartj/ehp249 19556262

[22] Camargo PR, Snitcowsky R, da Luz PL, Mazzieri R, Higuchi ML, Rati M, et al Favorable effects of immunosuppressive therapy in children with dilated cardiomyopathy and active myocarditis. Pediatric Cardiology. 1995;16(2):61–8. 10.1007/BF00796819 7784236

[23] Frustaci A, Chimenti C, Pieroni M, Salvatori L, Morgante E, Sale P, et al Cell death, proliferation and repair in human myocarditis responding to immunosuppressive therapy. Modern Pathology. 2006;19:755 10.1038/modpathol.3800594 16575400

[24] Ibrahim YF, Shults NV, Rybka V, Suzuki YJ. Docetaxel Reverses Pulmonary Vascular Remodeling by Decreasing Autophagy and Resolves Right Ventricular Fibrosis. The Journal of pharmacology and experimental therapeutics. 2017;363(1):20–34. 10.1124/jpet.117.239921 28760737 PMC5596829

[25] Wang X, Ibrahim YF, Das D, Zungu-Edmondson M, Shults NV, Suzuki YJ. Carfilzomib reverses pulmonary arterial hypertension. Cardiovasc Res. 2016;110(2):188–99. 10.1093/cvr/cvw047 26952044 PMC4836627

[26] Albini A, Pennesi G, Donatelli F, Cammarota R, De Flora S, Noonan DM. Cardiotoxicity of anticancer drugs: the need for cardio-oncology and cardio-oncological prevention. Journal of the National Cancer Institute. 2010;102(1):14–25. 10.1093/jnci/djp440 20007921 PMC2802286

[27] Sun X-Q, Kurakula K, Happé C, da Silva Goncalves Bos D, Schalij I, Vonk-Noordegraaf A, et al The effect of 6-mercaptopurine treatment on experimentally induced pulmonary arterial hypertension. European Respiratory Journal. 2017;50(suppl 61):OA4660.

[28] Mercaptopurine (Rx) [updated February 2019; cited 2019 9/11]. In Medscape—Drugs & Diseases. Available from: https://reference.medscape.com/drug/purinethol-purixan-mercaptopurine-342094.

[29] Andersen S, Schultz JG, Holmboe S, Axelsen JB, Hansen MS, Lyhne MD, et al A Pulmonary Trunk Banding Model of Pressure Overload Induced Right Ventricular Hypertrophy and Failure. Journal of visualized experiments: JoVE. 2018(141). 10.3791/58050 30582605

[30] Borgdorff MAJ, Dickinson MG, Berger RMF, Bartelds B. Right ventricular failure due to chronic pressure load: What have we learned in animal models since the NIH working group statement? Heart Failure Reviews. 2015;20(4):475–91. 10.1007/s10741-015-9479-6 25771982 PMC4463984

[31] Stepnowska E, Lewicka E, Dabrowska-Kugacka A, Miekus P, Raczak G. Prognostic factors in pulmonary arterial hypertension: Literature review. Advances in clinical and experimental medicine: official organ Wroclaw Medical University. 2017;26(3):549–53. 28791832 10.17219/acem/61855

[32] Vonk-Noordegraaf A, Haddad F, Chin KM, Forfia PR, Kawut SM, Lumens J, et al Right heart adaptation to pulmonary arterial hypertension: physiology and pathobiology. J Am Coll Cardiol. 2013;62(25 Suppl):D22–33. 10.1016/j.jacc.2013.10.027 24355638

[33] Lowes BD, Minobe W, Abraham WT, Rizeq MN, Bohlmeyer TJ, Quaife RA, et al Changes in gene expression in the intact human heart. Downregulation of alpha-myosin heavy chain in hypertrophied, failing ventricular myocardium. The Journal of clinical investigation. 1997;100(9):2315–24. 10.1172/JCI119770 9410910 PMC508428

[34] Bogaard HJ, Abe K, Vonk Noordegraaf A, Voelkel NF. The right ventricle under pressure: cellular and molecular mechanisms of right-heart failure in pulmonary hypertension. Chest. 2009;135(3):794–804. 10.1378/chest.08-0492 19265089

[35] Zungu-Edmondson M, Shults NV, Wong C-M, Suzuki YJ. Modulators of right ventricular apoptosis and contractility in a rat model of pulmonary hypertension. Cardiovascular Research. 2016;110(1):30–9. 10.1093/cvr/cvw014 26790474 PMC4798045

[36] Okada M, Harada T, Kikuzuki R, Yamawaki H, Hara Y. Effects of telmisartan on right ventricular remodeling induced by monocrotaline in rats. Journal of pharmacological sciences. 2009;111(2):193–200. 10.1254/jphs.09112fp 19809219

[37] Nagaya N, Nishikimi T, Uematsu M, Satoh T, Kyotani S, Sakamaki F, et al Plasma brain natriuretic peptide as a prognostic indicator in patients with primary pulmonary hypertension. Circulation. 2000;102(8):865–70. 10.1161/01.cir.102.8.865 10952954

[38] Schultz JG, Andersen S, Andersen A, Nielsen-Kudsk JE, Nielsen JM. Evaluation of cardiac electrophysiological properties in an experimental model of right ventricular hypertrophy and failure. Cardiology in the young. 2016;26(3):451–8. 10.1017/S1047951115000402 25872028

[39] Lopez B, Gonzalez A, Hermida N, Valencia F, de Teresa E, Diez J. Role of lysyl oxidase in myocardial fibrosis: from basic science to clinical aspects. American journal of physiology Heart and circulatory physiology. 2010;299(1):H1–9. 10.1152/ajpheart.00335.2010 20472764

[40] Smith-Mungo LI, Kagan HM. Lysyl oxidase: properties, regulation and multiple functions in biology. Matrix biology: journal of the International Society for Matrix Biology. 1998;16(7):387–98. 9524359 10.1016/s0945-053x(98)90012-9

[41] López B, González A, Lindner D, Westermann D, Ravassa S, Beaumont J, et al Osteopontin-mediated myocardial fibrosis in heart failure: a role for lysyl oxidase? Cardiovascular Research. 2013;99(1):111–20. 10.1093/cvr/cvt100 23619422

[42] Daniels A, van Bilsen M, Goldschmeding R, van der Vusse GJ, van Nieuwenhoven FA. Connective tissue growth factor and cardiac fibrosis. Acta physiologica (Oxford, England). 2009;195(3):321–38. 10.1111/j.1748-1716.2008.01936.x 19040711

[43] Rain S, Andersen S, Najafi A, Gammelgaard Schultz J, da Silva Gonçalves Bós D, Handoko ML, et al Right Ventricular Myocardial Stiffness in Experimental Pulmonary Arterial Hypertension. Relative Contribution of Fibrosis and Myofibril Stiffness. 2016;9(7). 10.1161/CIRCHEARTFAILURE.115.002636 PMC4956674 27370069

[44] Mendes-Ferreira P, Santos-Ribeiro D, Adao R, Maia-Rocha C, Mendes-Ferreira M, Sousa-Mendes C, et al Distinct right ventricle remodeling in response to pressure overload in the rat. American journal of physiology Heart and circulatory physiology. 2016;311(1):H85–95. 10.1152/ajpheart.00089.2016 27199115

[45] Egemnazarov B, Crnkovic S, Nagy BM, Olschewski H, Kwapiszewska G. Right ventricular fibrosis and dysfunction: Actual concepts and common misconceptions. Matrix biology: journal of the International Society for Matrix Biology. 2018;68–69:507–21. 10.1016/j.matbio.2018.01.010 29343458

[46] Esfandiary A, Kutsche HS, Schreckenberg R, Weber M, Pak O, Kojonazarov B, et al Protection against pressure overload-induced right heart failure by uncoupling protein 2 silencing. Cardiovascular Research. 2019;115(7):1217–27. 10.1093/cvr/cvz049 30850841 PMC6529920

[47] Cheng TC, Philip JL, Tabima DM, Hacker TA, Chesler NC. Multiscale structure-function relationships in right ventricular failure due to pressure overload. American journal of physiology Heart and circulatory physiology. 2018;315(3):H699–h708. 10.1152/ajpheart.00047.2018 29882684 PMC6172642

[48] Cartledge JE, Kane C, Dias P, Tesfom M, Clarke L, McKee B, et al Functional crosstalk between cardiac fibroblasts and adult cardiomyocytes by soluble mediators. Cardiovasc Res. 2015;105(3):260–70. 10.1093/cvr/cvu264 25560320

[49] Fujiu K, Nagai R. Fibroblast-mediated pathways in cardiac hypertrophy. J Mol Cell Cardiol. 2014;70:64–73. 10.1016/j.yjmcc.2014.01.013 24492068

[50] Zhou H, Wang J, Zhu P, Zhu H, Toan S, Hu S, et al NR4A1 aggravates the cardiac microvascular ischemia reperfusion injury through suppressing FUNDC1-mediated mitophagy and promoting Mff-required mitochondrial fission by CK2alpha. Basic research in cardiology. 2018;113(4):23 10.1007/s00395-018-0682-1 29744594

[51] Zheng J, Wei C-C, Hase N, Shi K, Killingsworth CR, Litovsky SH, et al Chymase Mediates Injury and Mitochondrial Damage in Cardiomyocytes during Acute Ischemia/Reperfusion in the Dog. PLOS ONE. 2014;9(4):e94732 10.1371/journal.pone.0094732 24733352 PMC3986229

[52] Cheng Z, Volkers M, Din S, Avitabile D, Khan M, Gude N, et al Mitochondrial translocation of Nur77 mediates cardiomyocyte apoptosis. European heart journal. 2011;32(17):2179–88. 10.1093/eurheartj/ehq496 21228009 PMC3164102

[53] Sun XQ, Abbate A, Bogaard HJ. Role of cardiac inflammation in right ventricular failure. Cardiovasc Res. 2017;113(12):1441–52. 10.1093/cvr/cvx159 28957536

[54] Braun MU, Szalai P, Strasser RH, Borst MM. Right ventricular hypertrophy and apoptosis after pulmonary artery banding: regulation of PKC isozymes. Cardiovasc Res. 2003;59(3):658–67. 10.1016/s0008-6363(03)00470-x 14499867

[55] Soon E, Holmes AM, Treacy CM, Doughty NJ, Southgate L, Machado RD, et al Elevated levels of inflammatory cytokines predict survival in idiopathic and familial pulmonary arterial hypertension. Circulation. 2010;122(9):920–7. 10.1161/CIRCULATIONAHA.109.933762 20713898

[56] Yoshida K, Abe K, Saku K, Sunagawa K. Inhibition of Nuclear Factor-kappaB-Mediated Inflammation Reverses Fibrosis and Improves RV Function in Rats with Pulmonary Artery Banding. Journal of Cardiac Failure.22(9):S198.

[57] Handoko ML, de Man FS, Happé CM, Schalij I, Musters RJP, Westerhof N, et al Opposite Effects of Training in Rats With Stable and Progressive Pulmonary Hypertension. Circulation. 2009;120(1):42–9. 10.1161/CIRCULATIONAHA.108.829713 19546388

[58] Handoko ML, de Man FS, Allaart CP, Paulus WJ, Westerhof N, Vonk-Noordegraaf A. Perspectives on novel therapeutic strategies for right heart failure in pulmonary arterial hypertension: lessons from the left heart. European respiratory review: an official journal of the European Respiratory Society. 2010;19(115):72–82. 20956170 10.1183/09059180.00007109 PMC9491638

[59] Ordentlich P, Yan Y, Zhou S, Heyman RA. Identification of the Antineoplastic Agent 6-Mercaptopurine as an Activator of the Orphan Nuclear Hormone Receptor Nurr1. Journal of Biological Chemistry. 2003;278(27):24791–9. 10.1074/jbc.M302167200 12709433

[60] Huang H-Y, Chang H-F, Tsai M-J, Chen J-S, Wang M-J. 6-Mercaptopurine attenuates tumor necrosis factor-α production in microglia through Nur77-mediated transrepression and PI3K/Akt/mTOR signaling-mediated translational regulation. Journal of neuroinflammation. 2016;13(1):78–. 10.1186/s12974-016-0543-5 27075886 PMC4831152

[61] Yoo YG, Na TY, Yang WK, Kim HJ, Lee IK, Kong G, et al 6-Mercaptopurine, an activator of Nur77, enhances transcriptional activity of HIF-1alpha resulting in new vessel formation. Oncogene. 2007;26(26):3823–34. 10.1038/sj.onc.1210149 17146432

[62] Lennard L. The clinical pharmacology of 6-mercaptopurine. European Journal of Clinical Pharmacology. 1992;43(4):329–39. 10.1007/bf02220605 1451710

[63] Cuffari C, Theoret Y, Latour S, Seidman G. 6-Mercaptopurine metabolism in Crohn's disease: correlation with efficacy and toxicity. Gut. 1996;39(3):401–6. 10.1136/gut.39.3.401 8949645 PMC1383347

[64] Gao X-F, Zhang J-J, Jiang X-M, Ge Z, Wang Z-M, Li B, et al Targeted drugs for pulmonary arterial hypertension: a network meta-analysis of 32 randomized clinical trials. Patient preference and adherence. 2017;11:871–85. 10.2147/PPA.S133288 28507431 PMC5428768

[65] Wu J, Bu L, Gong H, Jiang G, Li L, Ma H, et al Effects of heart rate and anesthetic timing on high-resolution echocardiographic assessment under isoflurane anesthesia in mice. Journal of ultrasound in medicine: official journal of the American Institute of Ultrasound in Medicine. 2010;29(12):1771–8. 10.7863/jum.2010.29.12.1771 21098849

