# Supplementary material for: Effects of 6-mercaptopurine in pressure overload induced right heart failure
Source: PLoS One. 2019 Nov 12;14(11):e0225122. doi: 10.1371/journal.pone.0225122 (PMC6850541; doi:10.1371/journal.pone.0225122)
Supplement: S1 Appendix — (DOCX) [file pone.0225122.s001.docx]

# **S1 Appendix**

Effects of 6-mercaptopurine in pressure overload

induced right heart failure

Julie Birkmose Axelsen^1^, Stine Andersen^1^, Xiao-Qing Sun^2^, Steffen Ringgaard^3^, Janus Adler Hyldebrandt^4^, Kondababu Kurakula^5^, Marie-José Goumans^5^, Frances S de Man^2^, Jens Erik Nielsen-Kudsk^1^, Harm-Jan Bogaard^2^, Asger Andersen^1^.

^1^Department of Cardiology – Research, Aarhus University Hospital, Denmark
^2^Department of Pulmonology, VU University Medical Center, Amsterdam
^3^MR Centre, Aarhus University Hospital, Denmark

^4^Department of Anesthesiology and Intensive Care, Aarhus University Hospital, Denmark

^5^Department of Cell and Chemical Biology, Leiden University Medical Center, Leiden, The Netherlands

## **Expanded methods**

## **Administration of 6-mercaptopurine and placebo**

To facilitate the dissolution of 6-MP in the drinking water, 6-MP was dissolved in dimethylsylfoxid (DMSO). The 6-MP-DMSO solution was stored at -20°C for a maximum of two weeks before renewing. Every morning the water bottles of the rats were weighed to measure water consumption during the past 24 hours. The rats were weighed, and the 6-MP dose was adjusted according to the weight of the heaviest rat in the cage and the amount of consumed water in the past 24 hours (ranged as 15-20-25-30-35 mL/day). Sham and placebo rats received placebo treatment consisting only of DMSO in the drinking water adjusted to the heaviest rat in the cage and consumed water the previous day in an equal manner.

## **Evaluation of hemodynamics**

### **Echocardiography**

Transthoracic echocardiography was performed under general anaesthesia (spontaneously breathing, sevoflurane 7% induction, 3.5% maintenance in 2:1 O_2_/air mix). The thorax of the rats was thoroughly shaved, and the remaining hair removed with Veet®. The scans were performed using a MS250 line array transducer on a Vevo 2100 imaging System (VisualSonics Inc., Toronto, ON, Canada) scanning at a frequency of 14-21 MHz. In an apical 4-chamber view, we measured tricuspid annular plane systolic excursion (TAPSE). Velocity time integral (VTI) in the pulmonary trunk was measured in the parasternal long axis view using the pulsed wave Doppler in the pulmonary trunk at three systematically chosen points, centrally and peripherally guided by colour Doppler. The systolic diameter of the pulmonary trunk was measured, and stroke volume (SV) computed as: $\text{SV=}\left( \frac{\text{PT diameter}}{\text{2}} \right)^{\text{2}}\text{∙π∙VTI}$. Heart rate was obtained through extremity electrodes. All parameters were measured in three consecutive heart cycles to average beat-to-beat variation. All images were analysed off-line (Vevo® 2100, Fujifilm VisualSonics Inc., Amsterdam, The Netherlands) with the observer blinded to the source of the sample. Echocardiography was performed 2 weeks after the PTB or sham procedure to obtain baseline results before initiating reversal- and placebo-treatment (table S1). Scans were performed again 7 weeks after the operation for final evaluation.

### **Magnetic resonance imaging**

A 9.4 Tesla Agilent MRI system was used to measure RV volume, ejection fraction, and cardiac output. Rats were anaesthetised (Sevoflurane 7% induction and 3.5% maintenance in 2:1 O_2_/N_2_O mix) and kept with spontaneous respiration during the scan. A volume transmit/receive rat coil was used. The imaging was synchronised to the heartbeat and respiration using a trigger system from SAII (New York, USA). For volume measurements, a set of short axis cine images were acquired covering the entire RV. Slice thickness was 1.5 mm and in-plane resolution was 0.2 mm. The data were evaluated using Segment v2.0 R5673 (http://segment.heiberg.se)(1). The RV endocardium was drawn by the automatic RV endocardium drawing function and afterward manually corrected during a full heart cycle on each slice, hereby obtaining values of end-systolic volumes (ESV) and end-diastolic volumes (EDV). For blood flow measurements in the pulmonary artery, a phase contrast flow measurement was obtained and flow values calculated using specially made analysis software (Siswin). Body surface-adjusted cardiac output, cardiac index (CI), was calculated as $\text{CI=CO/body surface area (BSA)}$, where $\text{BSA=}\text{body weigh}\text{t}^{\frac{\text{2}}{\text{3}}}\text{/100}$. All analyses were performed with the observer blinded to the source of the images.

### **Invasive pressure-volume measurements**

The rats were anesthetized with sevoflurane (7% induction, 3.5% maintenance in 2:1 O_2_/air mix), intubated, ventilated (Abbot Scandinavia, Solona, Sweden; RF 76 min^-1^ and tidal volume 4.5 mL), and injected with 50 units of heparin i.m. (Heparin, Leo Pharma A/S, Ballerup, Denmark) to prevent blood clotting during the procedure. Systemic blood pressures were measured by a pressure catheter (SPR-320, Millar Instruments, Houston, TX) installed in the left carotid artery after stabilization. RV pressure-volume loops were obtained using a conductance catheter (SPR-869, Millar Instruments, Houston, TX) installed in the RV through the apex. Signals were sampled by MPVS Ultra (Millar Instruments) and processed in Powerlab 16/35 (AD Instruments, UK). After stabilization, steady state RV pressures were recorded. Slowly occlusion of the inferior vena cava allowed simultaneous recordings of RV pressures and volumes with decreasing preloads, producing consecutive pressure-volume loops. Load-independent measures of RV contractility, including end-systolic elastance (Ees) and ventriculo-arterial coupling, and diastolic function were calculated from the pressure-volume loops using LabChart Software (AD Instruments, UK). The conductance signal was calibrated, using ESV and EDV derived from MRI volume measurements.

## **Cardiac histology and molecular analyses**

### **Histology**

Cardiac tissue was immersion fixated in formalin 4%, transferred to PBS after 36 hours, and subsequently embedded in paraffin. The cardiac tissue was cut in 5 µm sections on a rotary microtome and stained with haematoxylin & eosin (HE) for determination of LV and RV cardiomyocyte cross sectional areas (CSA). Cardiomyocyte CSA for each ventricle was calculated in ImageJ (Rasband, W.S., ImageJ, U. S. National Institutes of Health, Bethesda, Maryland, USA, https://imagej.nih.gov/ij/, 1997-2016) and expressed as the average CSA of minimum fifteen randomly distributed cardiomyocytes transversally cut at the level of the nuclei.

Another set of sections were stained with collagen specific picrosirius red to visualize fibrosis. RV fibrosis was expressed as the mean percentage tissue area positive for collagen measured over minimum six randomly chosen areas per ventricle. The percentage was calculated by adjusting collar thresholds in ImageJ. Areas with larger vessels and contaminations were excluded.

All histological analyses were performed by an operator blinded to the clinical source of the samples.

### **Polymerase chain reaction**

For investigation of underlying molecular mechanisms, the mRNA expression levels of genes involved in cardiac remodelling were evaluated by real-time polymerase chain reaction (PCR) analyses. A commercial RNA purification kit (RNeasy® Mini kit, Qiagen) was used to isolate RNA from 30-40 mg of snap-frozen RV tissue according to manufactures instructions. The RNA concentration in each sample was determined using a spectrophotometer (Eppendorf® BioPhotometer). The samples were diluted in RNase free H_2_O to contain 1 µg RNA in 15 mL fluid in total. Total RNA was reverse transcribed into complimentary DNA (cDNA) using iScript^tm^ cDNA Synthesis Kit (Bio-Rad) following a standard protocol. First strand cDNA was diluted x20 and real-time PCR was performed using iQ SYBR Green qPCR supermix (Bio-Rad), CFX96 Real-Time System (Bio-Rad) along with specific primers for the following genes: collagen 1, collagen 3a, osteopontin 1, connecting tissue growth factor (CTGF), lysyl oxidase (LOX), fibronectin 1 (FN1), brain natriuretic peptide (BNP), myosin heavy chain-β (MHC-β), interleukin 6 (IL-6), monocyte chemotactic protein 1 (MCP-1), and Nur77 (Table A). The housekeeping gene GAPDH was used for normalization. Collagen 1/3a ratio was calculated for each sample.

**Table A. Primer sequences for real-time polymerase chain reaction**

| Primer sequences | Forward | Reverse |
| --- | --- | --- |
| Collagen 1 | GAACGGAGATGATGGGGAAG | CCAAACCACTGAAACCTCTG |
| Collagen 3a | AGTGGCCATAATGGGGAACG | CAGGGTTTCCATCCCTTCCG |
| Osteopontin 1 | CCCATCTCAGAAGCAGAATCTT | GTCATGGCTTTCATTGGAGTTG |
| CTGF | CTGTTCCAAGACCTGTGGGAT | TTTTGCCCTTCTTAATGTTCT |
| LOX | GCCTGGACCGTGCTCTTTCT | CGTTGTTCTCCCATTGGATTGT |
| FN1 | CCAGGCACTGACTACAAGAT | CATGATACCAGCAAGGAGTT |
| BNP | GCTGCTTTGGGCAGAAGATAGA | GCCAGGAGGTCTTCCTAAAACA |
| MHC-β | ACCGTCAACACCAAGAGGGTCA | TTGGATGATTTGATCTTCCAAGG |
| IL-6 | TGTTCTCAGGGAGATCTTGG | TCCAGGTAGAAACGGAACTC |
| MCP-1 | CAGATCTCTCTTCCTCCACCACTAT | CAGGCAGCAACTGTGAACAAC |
| Nur77 | TGTTGCTAGAGTCCGCCTTT | CAGTGATGAGGACCAGAGCA |
| GAPDH | TAAAGGGCATCCTGGGCTACACT | TTACTCCTTGGAGGCCATGTAGG |

CTGF: connective tissue growth factor; LOX: lysyl oxidase; FN1: fibronectin 1; BNP: brain natriuretic peptide; MHC- β: myosin heavy chain- β; IL-6: interleukin 6; MCP-1: monocyte chemotactic protein 1; GAPDH: glyceraldehyde 3-phosphate dehydrogenase.

### **Nuclear and cytoplasmic fractioning**

Nuclear and cytoplasmic fractioning of tissue lysates were prepared and western blotting was performed as described previously (2, 3). Briefly, the tissues were lysed in 75 µL of buffer A (20 mmol/L Hepes pH 8, 10 mmol/L KCl, 0.15 mmol/L EGTA, 0.15 mmol/L EDTA, 0.15 mmol/L spermidine, 0.15 mmol/L spermine, 0.5 mmol/L Na_3_VO_4_, 5 mM NaF, 1 mmol/L DTT, 0.1% NP-40) supplemented with 1 mmol/L PMSF and protease inhibitor cocktail (Roche) for 10 minutes on ice. Next 15 µL of Sucrose Buffer (50 mmol/L Hepes pH 7, 0.25 mmol/L EDTA, 10 mmol/L KCl and 70% sucrose) was added and centrifuged at 5 x 10^3^ rpm for 10 minutes. The supernatant was used as the cytosolic fraction, which was subsequently used in the western blot analysis as described below. For obtaining the nuclear fraction, the pellet was washed in Buffer B (20 mmol/L Hepes pH 8, 50 mmol/L NaCl, 0.15 mmol/L EGTA, 0.25 mmol/L EDTA, 1.5 mmol/L DTT, 1.5 mmol/L MgCl_2_, 25% glycerol) supplemented with 1 mmol/L PMSF and protease inhibitor cocktail. The samples were centrifuged for 5 minutes at 5 x 10^3^ rpm. Then the pellet was resuspended in 100 µL Buffer C (Buffer B with 400 mmol/L NaCl), followed by incubation on ice for 30 minutes. After incubation, samples were centrifuged for 5 minutes at high speed, and the supernatant was collected, which was considered as the nuclear fraction, and used in the subsequent western blot analysis. Nuclear and cytoplasmic fractions were separated by SDS-PAGE and transferred onto nitrocellulose membranes (Millipore). After protein transfer, membranes were blocked with Tris-Buffered Saline solution containing 0.1% Tween 20 and 5% BSA for 1 hour at room temperature, and incubated with the appropriate primary antibodies and horseradish peroxidase-conjugated antimouse or anti-rabbit (GE Healthcare) secondary antibodies, followed by scanning using an ECL system (Fisher Scientific). Antibodies applied in this study were Nur77 (1:1000; Abcam), PCNA (1:1000; Sigma), GAPDH (1:1000; Millipore) and Vinculin (1:1000 dilution; H300; Santa Cruz).

### **Western Blot**

A downstream molecule of 6-MP was assessed together with inflammation and apoptosis markers using western blots with specific antibodies against Nur77 (1:1.000, ab109180, Abcam, Great Britain), CD45 (1:500, sc-53045, Santa Cruz, USA), and cleaved caspase 3 (1:1000, Asp175, Cell Signalling Technology, USA). Protein was extracted from 50-100 mg snap-frozen RV tissue, homogenized in homogenization buffer (PMSF (100x), Phospho 2, Phospho 3, RIPA-buffer (Sigma), Complete mini protease inhibitor), and centrifuged for 30 min followed by isolation of the supernatants. The samples were diluted 1:20 in PBS, and the protein concentrations were determined by Pierce 600 nm protein assay kit (Thermo Scientific, Pierce Biotehcnology, Rockford, USA). 20 µg of protein was used to detect the target protein expression. Equal sample loadings were separated on Invitrogen Novex Nupage Bis-tris Gel 4-12% (Thermo Fisher) and transferred to Amersham Protran Premium 0.45 NC Nitrocellulose Western Blotting Membranes (GE Healthcare Life Science) and blocked with 5% BSA-NaN3. The membranes were incubated with primary antibody at 4°C overnight followed by secondary antibody for 1-2 hours at room temperature. Before and after each incubation the membranes were washed 3x5 min with TBST. The blots were developed with Amersham ECL Prime Western Blotting Detection Reagent (GE Healthcare Life Science), and the membranes were scanned and analysed using Odyssey Infrared Imaging System (Li-COR Biosciences). GAPDH (1:50.000, G9295, Sigma-Aldrich, St. Louis, USA) or beta actin (1:50.000, A3854, Sigma-Aldrich, St. Louis, USA) were used for normalization.

### **Immunofluorescence Staining**

Cardiac paraffin sections were deparaffinised, rehydrated, boiled 40 min in Vector® Antigen Unmasking Solution, and blocked with 1% BSA for 2 hours at room temperature. The sections were incubated overnight at 4°C with primary antibody against Nur77 (1:50; sc-5559, Santa Cruz). Afterwards the sections were mounted with secondary antibody Nur77 (1:200; anti-rabbit FITC 488, Invitrogen, MA) containing DAPI (nuclei) followed by WGA counterstaining (1:50; Cy3 555; glycocalyx).

## **Reference list**

1. Heiberg E, Sjogren J, Ugander M, Carlsson M, Engblom H, Arheden H. Design and validation of Segment--freely available software for cardiovascular image analysis. BMC medical imaging. 2010;10:1.

2. Sanchez-Duffhues G, de Vinuesa AG, Lindeman JH, Mulder-Stapel A, DeRuiter MC, Van Munsteren C, et al. SLUG is expressed in endothelial cells lacking primary cilia to promote cellular calcification. Arteriosclerosis, thrombosis, and vascular biology. 2015;35(3):616-27.

3. Kurakula K, Vos M, Logiantara A, Roelofs JJ, Nieuwenhuis MA, Koppelman GH, et al. Nuclear Receptor Nur77 Attenuates Airway Inflammation in Mice by Suppressing NF-kappaB Activity in Lung Epithelial Cells. Journal of immunology (Baltimore, Md : 1950). 2015;195(4):1388-98.

## **Supplementary results**

## **Table B: Baseline echocardiography data**

| **Echocardiography data** | **Control** | **PTB** | | |
| --- | --- | --- | --- | --- |
|  | Sham  n=10 | PTB-control  n=9 | PTB-prevention  n=10 | PTB-reversal  n=9 |
| HR (bpm) | 347±10 | 302±8** | 314±5 | 319±12 |
| TAPSE (mm) | 2.66±0.07 | 2.12±0.08*** | 2.22±0.11 | 2.14±0.08 |
| CO (mL/s) | 153±18 | 134±7 | 152±9 | 136±11 |
| SV (mL) | 0.45±0.06 | 0.45±0.02 | 0.48±0.03 | 0.42±0.03 |

HR: Heart rate. TAPSE: Tricuspidal annular plane systolic excursion. CO: cardiac output. SV: Stroke volume.

Data are presented as mean ± SEM.

**P<0.01; ***P<0.001 vs sham
